# Fine-scale movement responses of free-ranging harbour porpoises to capture, tagging and short-term noise pulses from a single airgun

**DOI:** 10.1098/rsos.170110

**Published:** 2018-01-10

**Authors:** Floris M. van Beest, Jonas Teilmann, Line Hermannsen, Anders Galatius, Lonnie Mikkelsen, Signe Sveegaard, Jeppe Dalgaard Balle, Rune Dietz, Jacob Nabe-Nielsen

**Affiliations:** Department of Bioscience, Aarhus University, Frederiksborgvej 399, Roskilde 4000, Denmark

**Keywords:** anthropogenic disturbance, cetaceans, movement, offshore energy, *Phocoena phocoena*, underwater noise

## Abstract

Knowledge about the impact of anthropogenic disturbances on the behavioural responses of cetaceans is constrained by lack of data on fine-scale movements of individuals. We equipped five free-ranging harbour porpoises (*Phocoena phocoena*) with high-resolution location and dive loggers and exposed them to a single 10 inch^3^ underwater airgun producing high-intensity noise pulses (2–3 s intervals) for 1 min. All five porpoises responded to capture and tagging with longer, faster and more directed movements as well as with shorter, shallower, less wiggly dives immediately after release, with natural behaviour resumed in less than or equal to 24 h. When we exposed porpoises to airgun pulses at ranges of 420–690 m with noise level estimates of 135–147 dB re 1 µPa^2^s (sound exposure level), one individual displayed rapid and directed movements away from the exposure site and two individuals used shorter and shallower dives compared to natural behaviour immediately after exposure. Noise-induced movement typically lasted for less than or equal to 8 h with an additional 24 h recovery period until natural behaviour was resumed. The remaining individuals did not show any quantifiable responses to the noise exposure. Changes in natural behaviour following anthropogenic disturbances may reduce feeding opportunities, and evaluating potential population-level consequences should be a priority research area.

## 1. Introduction

The encroachment of anthropogenic disturbance into wildlife habitats is expanding at an unprecedented rate with potential implications for successful conservation of many species worldwide [1–3]. In marine habitat, a major and growing source of disturbance for wildlife is underwater noise emitted during offshore human activities [4–7]. Cetaceans (whales, dolphins and porpoises) are believed to be particularly sensitive to underwater noise because of their dependency on sound for effective communication, navigation and foraging [8,9]. Increased underwater noise levels may impact the physiology [10,11], hearing capacity [12,13] and/or behaviour [14,15] of cetaceans. Understanding individual-based behavioural responses to anthropogenic noise sources is a first and essential step towards estimating potential consequences on individual fitness and ensuing effects on population size and dynamics [16,17].

Some of the most intense underwater noise pulses are emitted during activities such as clearance of unexploded ordnance, pile driving during offshore construction, military sonar exercises and seismic survey operations using airgun arrays [8,18–20]. Most of the current knowledge on the effect of high-intensity underwater noise pulses on the movement behaviour of free-ranging individuals is based on large cetacean species as measured during authentic military sonar exercises [21–24] and seismic surveys [25,26]. For smaller cetacean species, however, much less is known about the influence of high-intensity impulsive noise on the movement behaviour of free-ranging individuals. To increase our understanding of the impact of underwater noise on the behavioural responses of smaller cetaceans detailed data on fine-scale movements of individuals (through biologging) in conjuncture with noise exposure experiments are required. However, capture and handling of wild animals to equip them with movement tags can also be considered a source of anthropogenic disturbance and care must be taken that any movement responses detected following noise exposure are unbiased by altered movement following capture/tagging activities.

The harbour porpoise (*Phocoena phocoena*) is the smallest and among the most common cetacean species inhabiting the European Atlantic shelf waters [27]. The species is protected throughout European waters and is listed in Annex II and IV of the European Union (EU) Habitats Directive [28]. Harbour porpoises typically prey on relatively small fish and must feed nearly continuously to support their high metabolic demands [29]. Abrupt changes in the natural behaviour of harbour porpoises could, therefore, elicit severe energetic costs making them highly susceptible to anthropogenic disturbances [29]. Indeed, previous studies have shown that high-intensity noise pulses emitted during authentic seismic surveys and pile driving activities can lead to marked declines in echolocation activity and buzz (feeding) rate [30,31] as well as temporary declines in local harbour porpoise density [32–34]. Nonetheless, the current evidence is based solely on passive acoustic monitoring and aerial survey data precluding the identification of the behavioural mechanisms underlying changes in local density within ensonified areas [32,34]. As a first step to fill this knowledge gap, we equipped five free-ranging harbour porpoises with high-resolution location and dive loggers and exposed them to short-term, high-intensity airgun noise pulses in their natural habitat. We then aimed to detect, quantify and compare the strength and duration of behavioural responses between two anthropogenic disturbances namely capture/tagging and underwater noise.

## 2. Material and methods

### 2.1. Study area

The study was conducted within the inner Danish waters, between Skagerrak in the north (58°00′ N) and the Belt Sea in the south (55°10′ N), where harbour porpoises are distributed widely with a relatively high abundance [35,36]. Most of the area has shallow water depths of 50 m but depths down to 500 m occur in the northern part of the area (electronic supplementary material, figure S1).

### 2.2. Harbour porpoise capture, handling and tagging

Five harbour porpoises were incidentally caught in pound nets between March and November 2014 (table 1; electronic supplementary material, figure S2A). Each porpoise was lifted out of the net and into a fishing boat by hand for tagging. Although we did not perform hearing tests (audiograms) on the captured individuals to keep the handling period and potential discomfort to a minimum, all five individuals were subjectively scored to be in good physical condition and large enough for tagging (minimum 120 cm standard length, table 1). We thus assumed the hearing abilities of the individuals were also good, as is generally the case for wild porpoises in this area [37]. Each individual porpoise was fitted with a custom-built ‘V-tag’ (electronic supplementary material, figure S2B) containing a very high frequency (VHF) radio transmitter (ATS, Isanti, MN, USA), an ARGOS transmitter (SPOT5, Wildlife Computers, Redmond, WA, USA), a Fastloc Global Positioning System (GPS) unit (F5G 133A, Sirtrack, Havelock North, New Zealand) and a time--depth recorder (TDR-unit; Lat1800ST, Lotek, Ontario, Canada or DST F-milli, StarOddi, Reykjavik, Iceland). The GPS-unit attempted to acquire and store a location every time the porpoise surfaced, while the TDR-unit registered the depth value every second. The V-tag was designed to detach after approximately 10 days. A more detailed description of the capture, handling and tag-fitting procedures is provided in the electronic supplementary material.

**Table 1.** Overview of individual, capture/tagging and noise-related information for each of the five harbour porpoises exposed to short-term pulses from a single airgun in the inner Danish waters between March and November 2014. Note that horizontal (location) movement data were missing for porpoise ID 5 and as such distance to exposure site could not be obtained and noise levels (given as *L*_pp_, *L*_eq-fast_ and SEL) could not be estimated. Date and time (local) are given as YYYY-MM-DD, HH:MM.

porpoise ID number	ID1	ID2	ID3	ID4	ID5
sex	male	male	male	female	female
body mass (kg)	31	30	37	30	36
standard length (cm)	122	143	143	136	122
mass/length index	0.25	0.21	0.26	0.22	0.29
location data available	yes	yes	yes	yes	no
ARGOS no.	2014-138066	2014-138072	2014-83307	2014-83303	2014-06421
dive data available	yes	yes	yes	no	yes
date and local time of porpoise release	2014-06-02, 12:00	2014-11-13, 13:25	2014-11-21, 11:30	2014-11-21, 11:38	2014-03-20, 13:40
date and local time of porpoise exposure	2014-06-05, 17:10	2014-11-17, 13:21	2014-11-25, 13:43	2014-11-26, 12:54	2014-03-24, 12:37
hours from release to exposure	77	96	98	121	95
distance (m [50% range]) from exposure	690 [345–1035]	610 [305–915]	420 [210–630]	550 [275–825]	—
Beaufort sea state during exposure	0	2	1	1	0
sediment type at exposure site	sand	diamicton	sand	sand	muddy sand
bathymetry (m) at exposure site	25.2	11.3	45.4	16.1	35.7
slope (°) of seabed at exposure site	0.9	0.3	0.7	0.2	0.14
porpoise depth (m) at start of exposure	13	0	0	—	0
mean porpoise depth (m) during exposure	13	5	14	—	2
*L*_pp_ (estimated mean [range] dB re 1 µPa peak–peak)	168 [165–173]	169 [166–174]	171 [168–177]	169 [166–175]	—
*L*_eq-fast_ (estimated mean [range] dB re 1 µPa over 125 ms)	147 [144–152]	148 [145–153]	150 [147–156]	148 [145–154]	—
SEL (estimated mean [range] dB re 1 µPa^2^s)	138 [135–143]	139 [136–144]	141 [138–147]	139 [136–145]	—
date and local time of tag release	2014-06-08, 03:11	2014-11-25, 12:00	2014-12-01, 20:00	2014-12-03, 23:11	2014-03-26, 06:44

### 2.3. Controlled exposure experiment and noise levels

Tagged porpoises were exposed to high-intensity noise pulses emitted by a single 10 inch^3^ underwater airgun (sleeve gun-I 40 inch^3^ with chamber inserts to reduce volume to 10 inch^3^; HGS, Halliburton Geophysical Services). Exposures were only conducted on days with a Beaufort sea state less than 3 (table 1), without rain and greater than or equal to 3 days after tagging. Three days was considered sufficient time to allow any capture/tagging-related movement behaviour to subside (we subsequently tested that presumption below). On the day of an exposure, the approximate location of a tagged porpoise was determined by online ARGOS satellite positions. With a custom-built aluminium research vessel (length of 6.6 m and width of 2.55 m and equipped with two 60 hp outboard engines), the porpoise was relocated further using VHF signals emitted by the tag (electronic supplementary material, figure S2C) and four 3-element yagi antennas and a DDF tracking unit with 360° diodes lighting in the direction of the animal. The research vessel used during the exposure experiments was not used during capture/tagging of the individuals and the individuals were, therefore, not expected to associate this vessel with any previous disturbance events. Once an individual porpoise was estimated to be approximately 500 m away from the research vessel (based on visual observations and/or VHF signal strength), the engines were shut down (i.e. the vessel was passively floating) and a single airgun was lowered into the water using a small, hand operated (non-hydraulic) crane (electronic supplementary material, figure S2D). When the airgun reached a depth of 7 m, single shots were fired at 2–3 s intervals (mean pressure of 125 bar per shot) for 1 min. The length of exposure, firing pressure and firing interval were limited by the amount of compressed air that could be transported on the research vessel.

**Figure 1.**
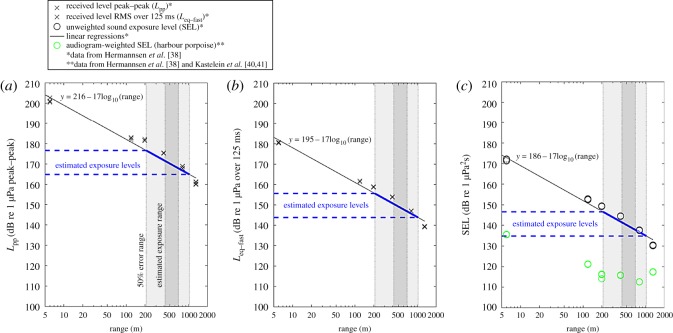
Estimated noise levels based on field recordings of a 10 inch^3^ airgun at high output pressure (120 bar) by Hermannsen *et al*. [38] shown as (*a*) received level peak–peak (*L*_pp_), (*b*) received level RMS over 125 ms (*L*_eq-fast_) and (*c*) sound exposure level (SEL). (*c*) Audiogram-weighted SELs using a harbour porpoise audiogram from Kastelein *et al*. [40,41]. The linear transmission loss model is given in each panel. Vertical grey bars show the estimated exposure range of the harbour porpoises included in this study incorporating a 50% error range in distance from the exposure site. The blue lines indicate the estimated range of noise exposure levels (*L*_pp_, *L*_eq-fast_ and SEL) experienced by the harbour porpoises in this study.

Noise levels of the airgun during the exposures were not recorded in the current study. Therefore, to estimate noise levels that porpoises were exposed to, we relied on field recordings from Hermannsen *et al*. [38] who studied the propagation of pulses emitted by the same 10 inch^3^ airgun at the same pressure (approx. 125 bar) and in a similar environment. These field recordings provided back-calculated source levels of 216 dB re 1 µPa_pp_ at 1 m (*L*_pp_, received sound pressure level peak to peak), 186 dB re 1 µPa^2^s at 1 m (sound exposure level, SEL, the pressure-squared time integral over a 1 s window around the pulse) and 195 dB re 1 µPa root mean squared (RMS) over 125 ms duration at 1 m (*L*_eq-fast_, approximating the auditory integration time of harbour porpoises [39]), and a transmission loss approximated by 17 log_10_(range), where range is in metres, for an area with a water depth of 15 m (figure 1). As the same airgun was used in this present study and the exposure experiments were conducted in similar shallow water environments (table 1), we assumed that noise levels and propagation of airgun pulses were similar to the characteristics of the 10 inch^3^ airgun recorded in Hermannsen *et al*. [38]. The exposure range, *r*, was the Euclidian distance (metres) between the exposure site and the position of the porpoise, calculated based on the GPS location of the airgun and the GPS location of the porpoise that was closest in time to the start of the exposure. However, due to variable time lags between successive GPS locations we could not determine the exact location of each individual at the start of the exposures. Therefore, the exposure ranges accommodated a 50% error in distance, as we expected the true location of the individuals during the exposures to be within this range. Including this 50% error range, the noise levels (*L*_pp_, *L*_eq-fast_ and SEL) of exposed individual porpoises were estimated to be in the ranges 165–177 dB re 1 µPa_pp_ (*L*_pp_), 144–156 dB re 1 µPa (*L*_eq-fast_) and 135–147 dB re 1 µPa^2^s (SEL) (table 1 and figure 1). As porpoises do not have equally sensitive hearing at all frequencies [40,41] SELs were frequency weighted with a harbour porpoise audiogram using the method from Hermannsen *et al*. [38], which gave an approximation of porpoises having experienced weighted SELs of 112–116 dB re 1 µPa^2^s (audiogram-weighted) resulting from the airgun exposures (figure 1).

### 2.4. Horizontal and vertical movement parameters

After the V-tags released from the individuals they were retrieved using positions from the ARGOS tag (long range) and VHF signal (short range). Movement data were obtained from five harbour porpoises, but partial tag failure resulted in missing vertical movement (TDR-unit) data for one individual (ID 4) and missing horizontal movement (GPS-unit) data for another (ID 5, table 1).

The GPS-units acquired, on average, one successful location out of four surfacing events. Location data were screened for positional outliers based on impossible movements, i.e. when the porpoise moved at an unlikely speed (greater than 15 km h^−1^) and returned to the same site it came from in the subsequent move. With this approach, we removed 112 locations (1.6% of the raw GPS dataset). After positional outliers were removed, we calculated for each location the displacement (kilometres) relative to release and the airgun exposure site as well as the compass heading of the individual. Displacement and compass heading were not analysed further using statistical modelling and served mainly as an independent procedure to assess the movement responses following capture/tagging and noise disturbance. We then continued by creating individual-specific horizontal movement trajectories using the ‘adehabitatLT’ package [42] in R (electronic supplementary material, figure S1). From each horizontal movement trajectory we extracted three parameters: step length (metres), speed of movement (m s^−1^) and absolute turning angles (0–180°) between relocations. Values close to 0° represent directed linear movement while values close to 180° represent tortious movements.

Preliminary screening and analyses of the TDR data revealed little drift in our pressure transducers and depth recordings, likely due to the relatively short attachment period (6−12 days, table 1). As such, we did not employ a zero-offset correction procedure. Data collected by the TDR-units were used to create individual-specific time--depth profiles using the ‘diveMove’ package [43] in R (electronic supplementary material, figure S3). From each time--depth profile, we extracted four vertical movement parameters: total dive duration (seconds) of each dive, maximum depth (metres) during each dive, wiggliness during each dive (as represented by the absolute vertical distance (metres) moved at the bottom phase of each dive, which is often used as an index of prey chasing behaviour or foraging intensity [21,24]) and post-dive duration (i.e. time (seconds) at the surface between dives).

### 2.5. Incorporating natural variation in movement behaviour

Natural variation in porpoise behaviour is substantial as movement and activity patterns typically differ between individuals [44], areas [36], seasons and diel time scales [45]. To account for natural variation in the movement data and to facilitate identification of any behavioural adjustments due to noise exposure, each movement parameter was regressed against hour of the day using generalized additive models (GAM), using the ‘mgcv’ package [46] in R [47]. To avoid any bias in our analyses due to lack of independence between the various movement parameters, a separate model was fitted for each movement parameter. Hour of the day was fitted as a cyclic regression spline, with the optimal smooth curve estimated by generalized cross-validation [46]. Because movement data are inherently autocorrelated [48,49], temporal dependence among observations was modelled using an autoregressive correlation structure (corAR1). A separate model was fitted for each individual to identify and account for individual-specific patterns in natural behaviour.

Results confirmed the presence of diel and individual variation in movement patterns (electronic supplementary material, figures S4–S7). To quantify baseline behaviour (i.e. the behavioural variation present after removing natural diel variation as much as possible), we extracted the raw residuals (data minus fitted values) of each GAM (i.e. at the conditional or individual level). We then calculated, for each individual separately, the mean value from all residuals within 1 h intervals over the complete tracking period. The 1 h time scale was used as this was the finest temporal scale we could attain without creating gaps (unbalance) in the horizontal movement data due to lack of GPS locations. In addition, by averaging the residual values in hourly intervals, we further reduced any temporal autocorrelation in the data. The hourly residuals provide a measure of deviation in horizontal or vertical movement patterns compared to the individuals' baseline behaviour at any given time (i.e. a residual value of 0). As an example, negative residual values for turning angles indicate more directed movements compared to the individuals' baseline behaviour, while positive residual values represent more tortious movements compared to the individuals' baseline behaviour.

### 2.6. Modelling movement responses to anthropogenic disturbances

**Figure 2.**
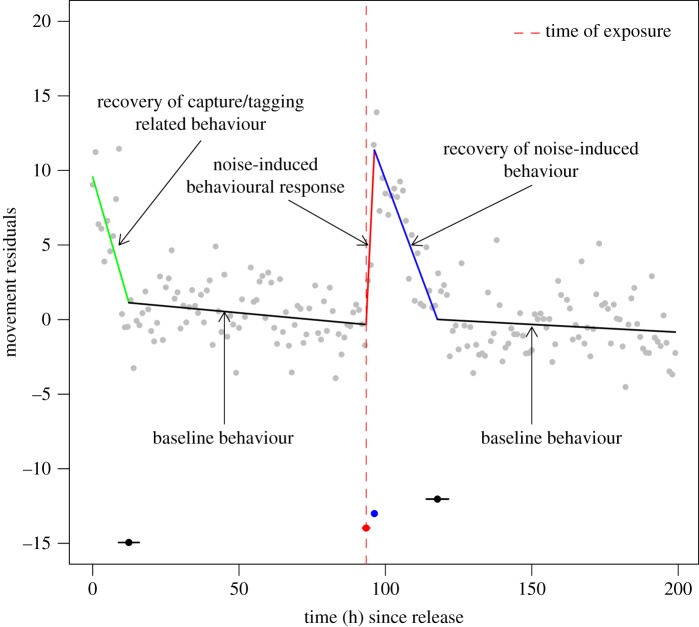
Illustration of a potential response to capture/tagging and short-term underwater noise for a given movement parameter (e.g. speed between GPS relocations) of a harbour porpoise individual as modelled with piecewise linear regression. The duration of the recovery of capture/tagging-related behaviour may be quantified by calculating the time difference between breakpoint 1 (black) and time of release (0 h). The duration of a noise-induced behavioural response may be quantified by calculating the time difference between breakpoint 3 (blue) and breakpoint 2 (red) but only if the second breakpoint (red) coincides with the time of exposure (vertical dashed line). The duration of the recovery of noise-induced behaviour may be quantified by calculating the time difference between breakpoint 4 (black) and breakpoint 3 (blue). Note that the slope of the regression lines may vary depending on the movement parameter analysed and the individual-specific response.

Movement responses of individual porpoises to anthropogenic disturbances (i.e. capture/handling and airgun noise exposure) were modelled with piecewise linear regression using the ‘segmented’ package [50] in R. This analytical framework is ideal when the dependent variable is expected to exhibit sudden shifts or divergent relationships with the independent variable, which are then identified by breakpoints [51,52]. Because marine species can change their behaviour instantaneously when exposed to anthropogenic disturbances such as underwater noise [21,22], piecewise regression is a suitable analytical approach to detect sudden changes in horizontal and vertical movement patterns of harbour porpoises over time. Assuming that harbour porpoises respond strongly to disturbance events such as capture/handling and short-term underwater noise, we would *a priori* expect to observe multiple shifts or changes in movement behaviour during the tracking period of each individual (figure 2). Specifically, we would expect movement patterns (e.g. speed) directly after the capture and tagging event to be distinctly different from baseline behaviour (i.e. natural movement) and to subside steadily over time until baseline behaviour was resumed, which is commonly observed following capture or tagging activities of marine species [53]. We call this the recovery period of capture and handling related behaviour (figure 2). We would also expect to observe a sudden shift in movement behaviour coinciding with the time of the airgun exposure event that differed from baseline behaviour (termed noise-induced behavioural response; figure 2). Noise-induced behaviour should, after some time, be followed by another (more gradual) change in movement until baseline behaviour was resumed (termed recovery period of noise-induced behaviour; figure 2). It is possible, however, that individuals display completely different behaviour or only some of the expected responses as demonstrated in figure 2. For example, individuals could display only recovery behaviour following capture and handling, or only noise-induced behavioural responses, or behavioural changes at times unrelated to the capture/handling and noise exposure events. We accommodated for all possible responses in our statistical procedure by fitting increasingly complex regression models and selecting the model that best fitted the data, as explained below.

The dependent variables in the piecewise regression models were the residuals of the GAM analyses for each movement parameter and the independent variable was time (hours) since release. This allowed us to formally compare the behavioural response effect of both capture/tagging and airgun noise (if detected), and to test if observed movements following the noise exposure were biased by long-lasting capture/tagging effects. This is important as the strength and duration of behavioural responses may vary between different types of disturbance as previously shown for other cetaceans [53]. We computed piecewise regression models for each individual separately to assess individual variation in behavioural responses to capture/tagging and the airgun noise.

For each movement parameter, we ran a series of regression models with variable number of breakpoints along the independent variable (time since release). We started off with a simple linear regression model without a breakpoint, suggesting no abrupt behavioural changes during the tracking period. We continued with fitting piecewise linear regression models to which we step-by-step introduced one additional breakpoint. Starting values of potential breakpoints were supplied by splitting up the tracking period into equally spaced periods (using the K parameter in the segmented package in R). We then ranked all models based on AIC_c_ values and selected the best fitting model for each movement parameter. We included a maximum of six breakpoints, as models with more breakpoints generally did not converge. Although equally spaced starting values for breakpoints were provided, the models were allowed sufficient tolerance to search for breakpoints with a potentially better fit along the complete time gradient. We evaluated the validity of each breakpoint by checking that the slope of the regression line before or after a breakpoint was significantly different from 0 (baseline behaviour), suggesting that a true change in behaviour was detected. To further increase confidence in the breakpoint values and to ensure that temporal autocorrelation did not bias the results (electronic supplementary material, figures S8–S9), we also calculated the bootstrapped 95% confidence interval (CI) for each accepted breakpoint by resampling the hourly residuals with replacement based on 50 iterations. Fifty bootstrap restarts are considered sufficient for most analyses with multiple breakpoints [50].

## 3. Results

### 3.1. Movement responses to capture/tagging

**Figure 3.**
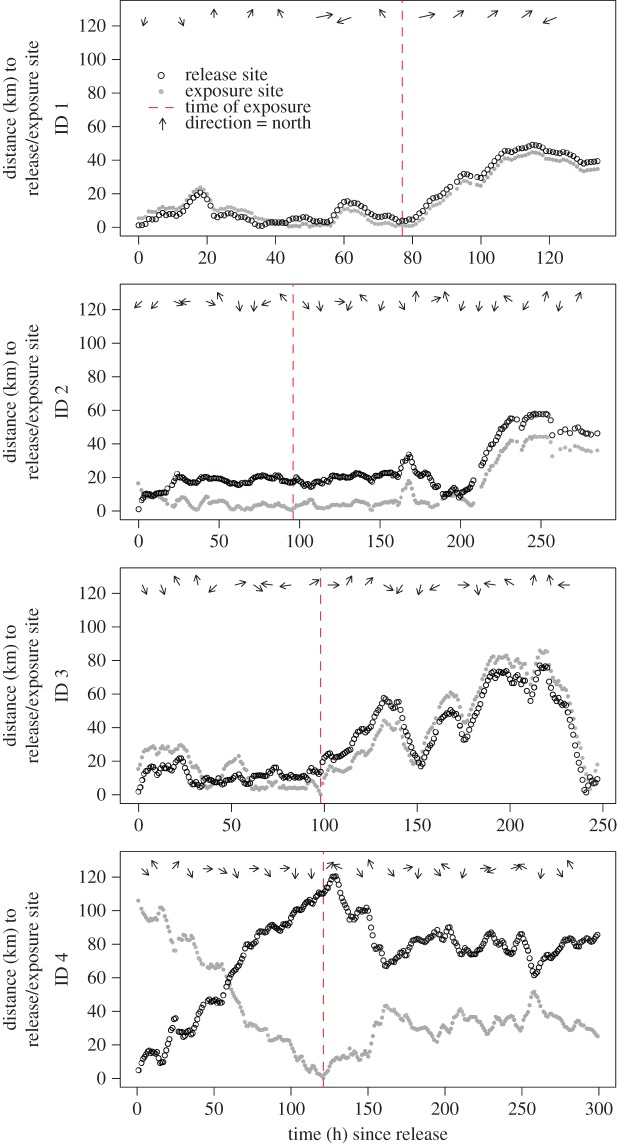
Distance (km) to the release and airgun exposure site over time for each individual harbour porpoise with location data available. Arrows indicate the compass direction of horizontal movements over 10 h intervals.

**Figure 4.**
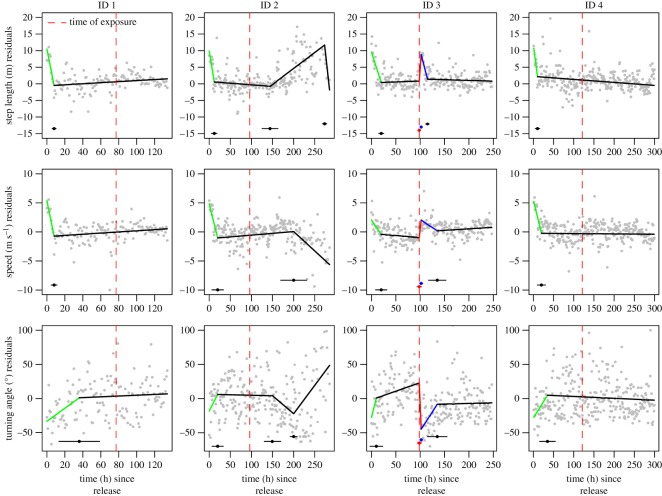
Results of piecewise regression models predicting breakpoints (mean and 95% CIs) in capture/tagging- and airgun exposure-related behaviour in three horizontal movement parameters for each individual harbour porpoise. Grey points are the residuals of the movement parameter. The residual values provide a measure of deviation in fine-scale horizontal movements compared to the individuals' baseline behaviour (i.e. a residual value of 0). Confidence intervals for regression lines are not drawn to improve visibility, but s.e. for each segment is provided in electronic supplementary material, table S2. Note that ID 5 did not have a functioning GPS-unit and could not be included in these analyses.

**Figure 5.**
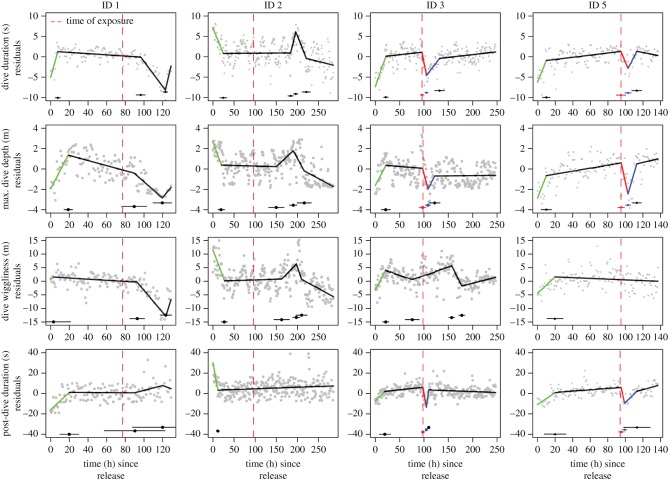
Results of piecewise regression models predicting breakpoints (mean and 95% CIs) in capture/tagging- and airgun exposure-related behaviour in four vertical movement parameters for each individual harbour porpoise. Grey points are the residuals of the movement parameter. The residual values provide a measure of deviation in fine-scale vertical movements compared to the individuals' baseline behaviour (i.e. a residual value of 0). Confidence intervals for regression lines are not drawn to improve visibility, but s.e. for each segment is provided in electronic supplementary material, table S2. Note that ID 4 did not have a functioning TDR-unit and as such could not be included in these analyses.

Results of the piecewise linear regression models suggested consistent and strong capture/tagging-related responses in horizontal and vertical movement patterns across all five porpoises (figures 3–5). Specifically, residuals of step length (metres) between relocations directly after release were typically 10 m longer than during baseline (figure 4). Step lengths (metres) subsequently declined steadily, until post-release behaviour had disappeared after approximately 12.5 h (mean of breakpoint 1 values across individuals for step length in electronic supplementary material, table S1). A similar pattern was observed for speed (m s^−1^) between relocations directly after release as porpoises moved approximately 5 m s^−1^ faster (an average increase of greater than 500%) than during baseline (figure 4; electronic supplementary material, figure S4). Speed of movement returned to baseline after approximately 17 h post-release (mean of breakpoint 1 values across individuals for speed in electronic supplementary material, table S1). Residuals of turning angles between relocations were largely negative directly after release, indicating directed horizontal movement away from the release site. Indeed, all four porpoises with functional GPS tags moved away from the capture/release site in a directed manner and covered 20 km in 20 h (figure 3). Turning angles between relocations normalized after approximately 25 h post-release (mean of breakpoint 1 values across individuals for turning angle in electronic supplementary material, table S1), though individual variation in the recovery period was substantial (figure 4).

Vertical movement patterns also showed strong capture/tagging-related effects (figure 5). Three out of four porpoises fitted with TDR tags (IDs 1, 3 and 5) used shorter (approx. 5 s), shallower (approx. 2 m), less wiggly dives (approx. 5 m) with shorter resting intervals (approx. 10 s) between dives directly after release as compared to baseline behaviour (figure 5; electronic supplementary material, figure S5). The recovery period of the capture-related vertical movement patterns was typically less than 20 h for these three individuals (figure 5; electronic supplementary material, table S1). Porpoise ID 2 showed the opposite response to capture/tagging activities as it used longer (approx. 7 s), deeper (approx. 3 m), wigglier dives (approx. 12 m) with longer resting intervals (approx. 25 s) directly after release (figure 5) than during its normal behaviour (electronic supplementary material, figure S5). Nonetheless, the recovery period of the capture-related vertical movement patterns was similar to those of other individuals with baseline behaviour attained within 27 h (electronic supplementary material, table S1).

Based on these results, all capture/tagging-related behavioural responses (in both horizontal and vertical movement parameters) were expected to have completely disappeared before the airgun exposure experiment was initiated (greater than or equal to 3 days after release).

### 3.2. Movement responses to single airgun noise exposure

Results of the piecewise linear regression models suggest that porpoise ID3 responded strongly to the noise exposure experiment that aligned with our expectations (figure 2). The individual displayed abrupt changes in both horizontal and vertical movement patterns that were triggered during the time of exposure (figures 4 and 5). We estimated that this individual also received the highest noise exposure level of all individuals (table 1). The noise-induced response for porpoise ID 3 comprised a temporary (4 h difference in time between breakpoint 3 and 2 provided in electronic supplementary material, table S1) increase in step length (approx. 10 m) and speed (approx. 2 m s^−1^) between relocations directly after exposure (figure 4) relative to its baseline behaviour (electronic supplementary material, figure S4). At the same time, it used directed horizontal movements (i.e. negative turning angle residuals, figure 4) away from the exposure site (figure 3), but returned to the exposure area after 6 days. Following the 4 h noise-induced response period, the horizontal movement patterns steadily returned to baseline behaviour, which was attained after approximately 35 h post-exposure (difference in time between breakpoint 4 and 3 provided in electronic supplementary material, table S1). Porpoise ID 3 also altered its vertical movement following noise exposure (figure 5) with temporary (8 h difference in time between breakpoint 3 and 2 provided in electronic supplementary material, table S1) declines in dive duration (approx. 5 s), maximum dive depth (approx. 2 m) and post-dive surface duration (approx. 15 s) as compared to baseline behaviour (electronic supplementary material, figure S5). We did not detect a change in dive wiggliness post-exposure for porpoise ID 3. As for the horizontal movements, dive behaviour steadily returned to baseline behaviour, which was attained after an additional 17 h post-exposure (difference in time between breakpoint 4 and 3 provided in electronic supplementary material, table S1).

Porpoise ID 5 had a malfunctioning GPS-unit, which precluded assessment of airgun-related changes in horizontal movement parameters and estimation of the noise exposure level (table 1). However, this individual did show clear and consistent airgun-related effects in vertical movement patterns that were similar in direction, strength and duration as observed for porpoise ID 3 (figure 5).

The piecewise linear regression models also identified multiple breakpoints in movement parameters for porpoise IDs 1, 2 and 4, suggesting abrupt changes in movement behaviour besides the capture/tagging responses, but these were unlikely to be airgun-related responses as the breakpoints did not centre on the individual-specific time of exposure (figures 4 and 5; electronic supplementary material, table S1).

## 4. Discussion

Studying the impact of anthropogenic disturbances on the behaviour of free-ranging individuals is inherently difficult due to logistical challenges with tracking movement of marine species during anthropogenic disturbance events. Here we present the first direct measurements of the fine-scale movement responses of individual harbour porpoises to two different types of disturbance events in their natural habitat, namely capture and tagging as well as short-term noise exposure by a single airgun. Behavioural responses to capture and tagging activities were detected across all individuals with site displacement and pronounced differences in both horizontal and vertical movement patterns, which lasted up to 24 h after which natural behaviour was resumed. Furthermore, and despite the short duration of the exposure (1 min) to a single, almost stationary, airgun, we found noise-induced behavioural responses by two of the five porpoises followed by a recovery period that was largely comparable to the capture and tagging responses in terms of duration. We also observed displacement movement away from the exposure area by one individual.

Our findings have important implications for future controlled exposure experiments, as well as more general movement ecology studies, of small cetacean species. It is imperative that any capture/tagging-related movement behaviour has disappeared before the noise exposure experiment is conducted. In our case, behavioural responses following capture and tagging activities had typically subsided 24 h after release, which was well before the exposure events took place (greater than or equal to 3 days after tagging). Clearly, the strength and duration of behavioural responses to capture, tagging or marking depend on the species and the methods used [54] but proper analysis of potential marking or tagging effects is needed to be confident that data collected from the study individuals are representative of natural behaviour prior to (experimental) disturbance events. This issue also applies to more general movement ecology studies without noise exposure experiments and we recommend that any location or dive data collected in the first 24 h after tagging are discarded or handled with care to ensure that potential capture/tagging-related effects on movement behaviour do not bias data analyses and conclusions.

Our study supports the notion that underwater noise can alter the movement behaviour of individual cetaceans [22–24,55,56]. Specifically, we extend current knowledge on the effect of high-intensity airgun noise pulses on the movement of individual harbour porpoises. Such high-intensity noise pulses with low-frequency emphasis that also contains considerable medium- to high-frequency noise are emitted during both authentic seismic surveys and pile driving activities. These anthropogenic marine activities have previously been linked to short-term (less than 1 day) reductions in echolocation activity and buzz (feeding) rate of harbour porpoises [30,31] as well as local density [32–34] but without obvious long-term (greater than 1 day) displacement effects [32]. Our results on individual-specific movements largely confirm these previous findings as we did not observe consistent and strong signals of site displacement following the underwater noise exposure, and behavioural alterations were typically short term (less than 2 days including the recovery period). We found these patterns despite major differences in the design and context of our exposure experiments compared to authentic seismic surveys as well as offshore pile driving activities. Our exposure experiments, for example, had only one airgun and did not have an acoustic ramp-up procedure or other warning signals (e.g. increased shipping activity) of an upcoming disturbance event as is obligatory prior to authentic seismic surveys and offshore pile driving activities (see [31,32] for details on authentic offshore pile driving operations and seismic surveys). As such, the movement patterns documented here may have been the result of individuals responding to a sudden and unexpected noise source that may not be generally applicable. This complexity should be considered carefully in interpreting and extrapolating our results. Similarly, DeRuiter *et al*. [22] also showed that noise-induced behavioural responses of Cuvier's beaked whales (*Ziphius cavirostris*) were less pronounced when exposed to distant but authentic military sonar signals compared to nearby simulated signals, despite similar received sound pressure levels. To what extent this phenomenon applies to harbour porpoises is unknown, highlighting a continued need to focus future behavioural studies on responses of free-ranging individuals to authentic seismic surveys or offshore pile driving operations. Importantly, we recommend that future underwater noise exposure studies should measure both the behavioural responses of individual animals (through e.g. biologging) and record the noise levels experienced by the study animal at each exposure site. Lack of acoustics data in our study limits inference about the exact sound levels that the porpoises responded to (or when they did not). It is possible to incorporate such acoustics data directly into our analytical approach to assess at what noise levels fine-scale movements of individuals start to deviate from natural behaviour. However, alternative statistical approaches to address this question are becoming available. For example, hierarchical hidden Markov models that can accommodate multiple data streams of different resolution have recently been developed to assess behavioural states from fine-scale movement data [57]. The advantage of such a time-series approach is that multiple movement parameters can be fitted jointly in one analysis so as to evaluate changes in behavioural states (e.g. resting, foraging, travelling) following anthropogenic disturbances.

Individual variability in responsiveness to human disturbance is frequently reported, and the animals' energetic status is thought to be an important determinant [6]. Individuals in poorer physical condition may be less likely to change their behaviour and displace from good foraging habitat following an acute disturbance event than individuals in optimal body condition, as the costs associated with reduced or missed feeding opportunities may be too high [58,59]. This theory is particularly relevant for harbour porpoises as they have a high metabolic demand and carry relatively small energy reserves compared to other cetacean species [29]. A short period without feeding (greater than 3 days) may rapidly reduce individual body mass and chances of survival [60,61]. The three porpoises that did not show any measurable responses to the airgun exposure experiment (IDs 1, 2 and 4) had the poorest physical condition (lowest mass/length index) of our tagged individuals (table 1). It is plausible that the energetic status of these individuals was at such low levels that they had a higher disturbance tolerance compared to the individuals that did respond to the exposure. Our sample size, however, is too small to perform a robust analysis of condition-, age- or sex-dependent responses to underwater noise.

Clearly, any disruption of natural behaviour or site displacement from important feeding areas due to anthropogenic disturbances conveys energetic costs through reduced or missed feeding opportunities [62]. Regular alterations in natural behaviour and/or displacement from high quality habitat may lead to declines or disruption in energy acquisition and negatively impact reproductive output with implications for population size and dynamics. Various simulation frameworks currently exist that aim to predict the impact of underwater noise on harbour porpoise individuals [63] and populations [64]. However, the behavioural responses to underwater noise by individuals incorporated into such models are largely based on movement theory and untested assumptions. Our empirical data and findings are valuable to improve such models as they provide a more detailed and solid basis for predictions on the strength and duration of the behavioural response of individuals to underwater noise. As such, a strong need remains to focus future wildlife-disturbance studies on bridging the knowledge gap between repeated short-term behavioural responses of individuals to anthropogenic disturbances and potential population-level consequences [17,64].

## Supplementary Material

Supporting figures, tables and detailed field protocol

## Data accessibility

Data are available from the Dryad Digital Repository: http://dx.doi.org/10.5061/dryad.40nv6 [65].
