# Supplementary material for: Fine-scale movement responses of free-ranging harbour porpoises to capture, tagging and short-term noise pulses from a single airgun
Source: R Soc Open Sci. 2018 Jan 10;5(1):170110. doi: 10.1098/rsos.170110 (PMC5792866; doi:10.1098/rsos.170110)
Supplement: Supporting figures, tables and detailed field protocol [file rsos170110supp1.docx]

**Electronic Supplementary Material (ESM)**

**Supplementary Information, Figures, and Tables**

**Fine-scale movement responses of free-ranging harbour porpoises to capture, tagging, and short-term noise pulses from a single airgun**

Floris M. van Beest, Jonas Teilmann, Line Hermannsen, Anders Galatius, Lonnie Mikkelsen, Signe Sveegaard, Jeppe Dalgaard Balle, Rune Dietz^,^ and Jacob Nabe-Nielsen


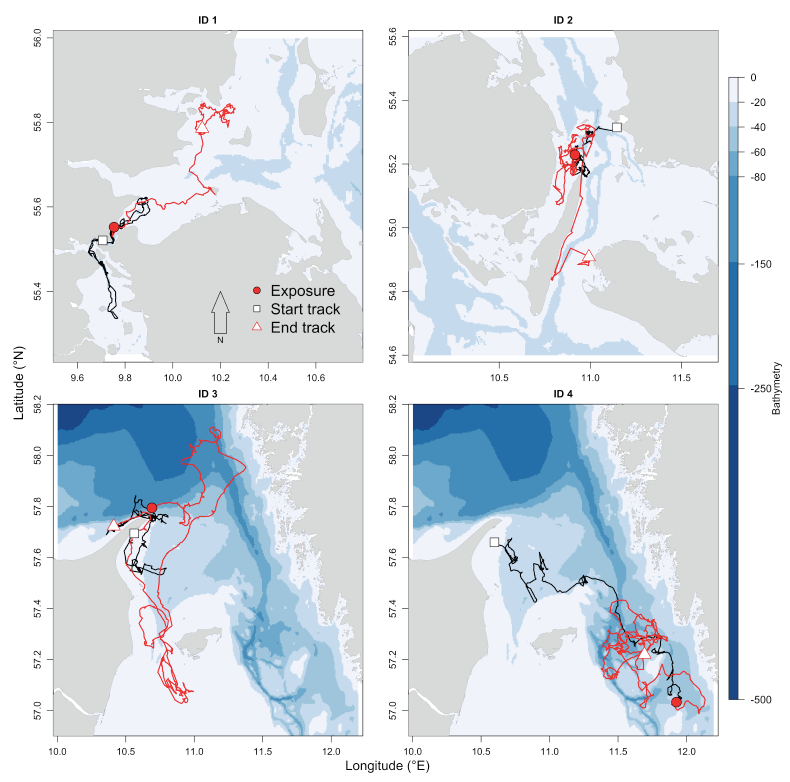


**Figure S1**. Horizontal movement trajectory of four harbour porpoises exposed to short-term noise pulses from a single airgun noise in the inner Danish waters. The location of the start of the trajectory (square), the end of the trajectory (triangle), and exposure site (circle) are shown for each porpoise. Black trajectory lines indicate horizontal movements prior to exposure and red trajectory lines indicate horizontal movements post exposure. Blue background colours indicate differences in bathymetry (m) and grey background colour indicates land. Note that porpoise ID 5 had a malfunctioning GPS-unit and as such the horizontal movement trajectory could not be plotted.

**Detailed description of harbour porpoise capture, handling, and tagging procedure**

Five harbour porpoises were caught and tagged in the study area between March−November 2014 (Table 1 in main article). Porpoises were caught incidentally in pound nets (Figure S2A). Pound nets are used in near-shore commercial fisheries in the inner Danish waters. They typically measure 10–30 m in diameter and 3–7 m in depth and have a small mesh size (2 × 2 cm). Pound nets pose no threat to the porpoises as they can breathe at the surface and swim freely while entrapped. Fishermen that encountered a porpoise in their nets contacted the research team immediately. Upon arrival of the research team, the fishermen would drag the net to the surface so that the porpoise could be lifted into the fishing boat by hand and placed on foam mattresses covered with a stretcher made of two poles and tarpaulin. Each porpoise was inspected for any physical damage or unusual appearance while breathing was monitored. A heart rate meter (Polar S810) was placed around the body behind the pectoral fins to monitor whether the heart rate remained between 50-200 beats per minute as recommended by Eskesen et al. [1]. During handling, all porpoises were continuously watered down to facilitate breathing and avoid overheating and drying of the skin.

When a caught individual was deemed large enough (min 120 cm standard length) and fit for tagging, it was fitted with two tags (Figure S2B). The first tag was called a V-tag and consisted of a hand-made high-density closed cell foam package containing a Fastloc Global Positioning System (GPS-unit; F5G 133A, Sirtrack, Havelock North, New Zealand) and a Time-Depth recorder/logger (TDR-unit; Lat1800ST, Lotek, Ontario, Canada or DST F-milli, StarOddi, Reykjavik, Iceland). The GPS-unit attempted to acquire and store a location every time the porpoise surfaced, while the TDR-unit registered a depth value every second using pressure transducers. Both the GPS and TDR data were used in this study. In addition to the GPS-, and TDR-units the V-tag contained a Very High Frequency (VHF) radio transmitter (ATS, Isanti, MN, USA) and a small ARGOS transmitter (SPOT5, Wildlife Computers, Redmond, WA, USA). The V-tag had a weight of 135−150g. The second tag was an ARGOS satellite transmitter (SPLASH or SPOT5, Wildlife Computers, Redmond, WA, USA), which was used to relocate the individuals prior to the exposure experiment. The ARGOS tags weighed 55g.

Both tags were attached to the porpoise by experienced researchers using published techniques [2]. The long-term ARGOS tag was mounted on the left side of the porpoise’s dorsal fin using two (SPOT5) or three (SPLASH) delrin 5 mm pins. The ends of the pins were used to attach the V-tag on the right side of the dorsal fin. The pins were designed to temporarily hold the V-tag in place using dissolving magnesium bolts so that the tag would release and drift to the surface after 10−14 days (Table 1 in main article).

All porpoises were tagged and released within 24 hours after being discovered in nets by fishermen. Total handling time of each porpoise during the tagging procedure was less than 30 min after which they were released back into the water. We did not experience any mortality or unexpected incidences during capture, handling or tagging of the porpoises.

All tagging and fieldwork activities, including the exposure experiment (see next section), performed during this study were carried out with permission of the Nature Agency (Danish Ministry of Environment, NST-3446-0016) and the Animal Experiments Inspectorate (Danish Ministry of Food, Agriculture and Fisheries, 2010/561-1801).

**References**

1. Eskesen IG, Teilmann J, Geertsen BM, Desportes G, Riget F, Dietz R, Larsen F, Siebert U. 2009 Stress level in wild harbour porpoises (*Phocoena phocoena*) during satellite tagging measured by respiration, heart rate and cortisol. *J. Mar. Biol. Assoc. U. K.* **89**, 885–892.

2. Teilmann J, Larsen F, Desportes G. 2007 Time allocation and diving behaviour of harbour porpoises (*Phocoena phocoena*) in Danish and adjacent waters. *J. Cetacean Res. Manage.* **9**, 201–210.


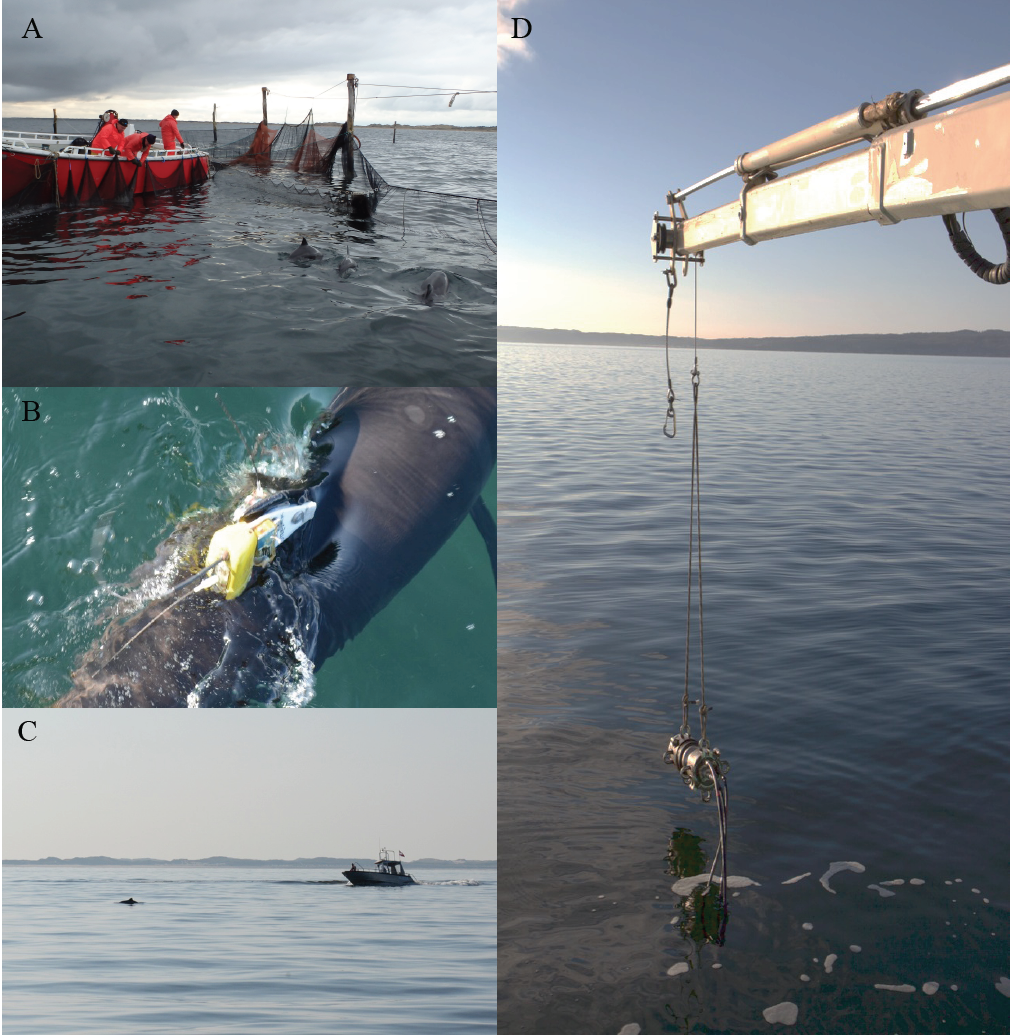


**Figure S2.** Overview of fieldwork protocol showing A) pound nets in which porpoises were incidentally caught, B) the V-tag attached to the dorsal fin, C) a research vessel tracking a tagged porpoise using VHF signals, and D) the 10 in^3^ airgun used during the exposure experiments. Note that the crane in Fig. S2D was not used during the exposures of individual porpoises, but instead a different non-hydraulic hand-operated crane was used on board of the research vessel.


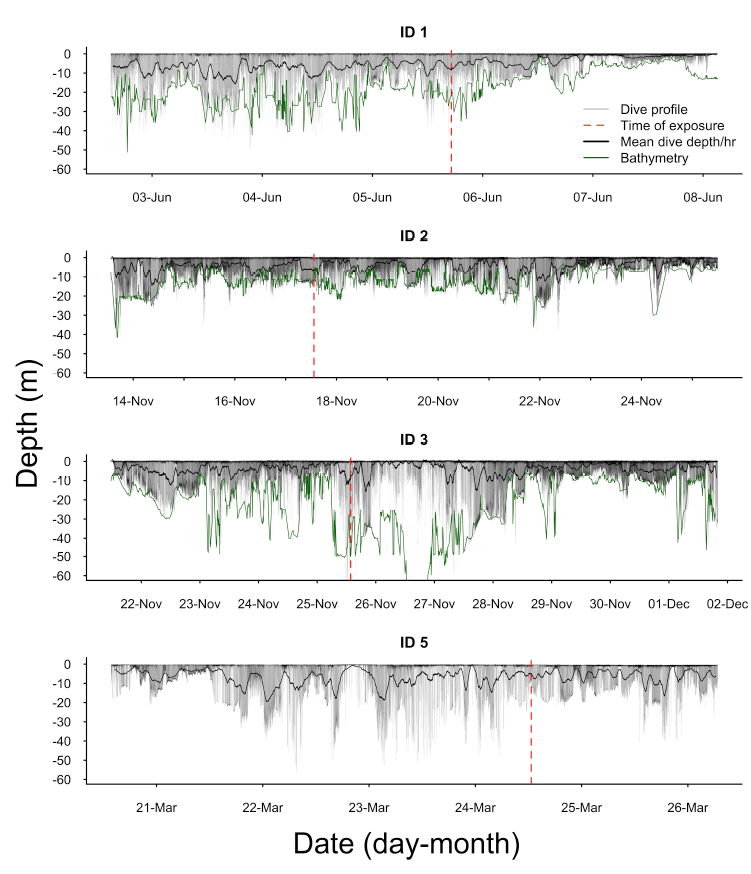


**Figure S3.** Complete dive profile of four harbour porpoises exposed to airgun pulses in the inner Danish waters. Individual dives (grey) are shown with the mean dive depth hr^-1^ (black) and time of exposure (red). Bathymetry (green) is based on the acquired GPS positions. Note that ID 4 did not have a functioning TDR-unit and as such the dive profile could not be plotted and ID 5 did not have a functioning GPS-unit and as such bathymetry could not be derived and plotted.


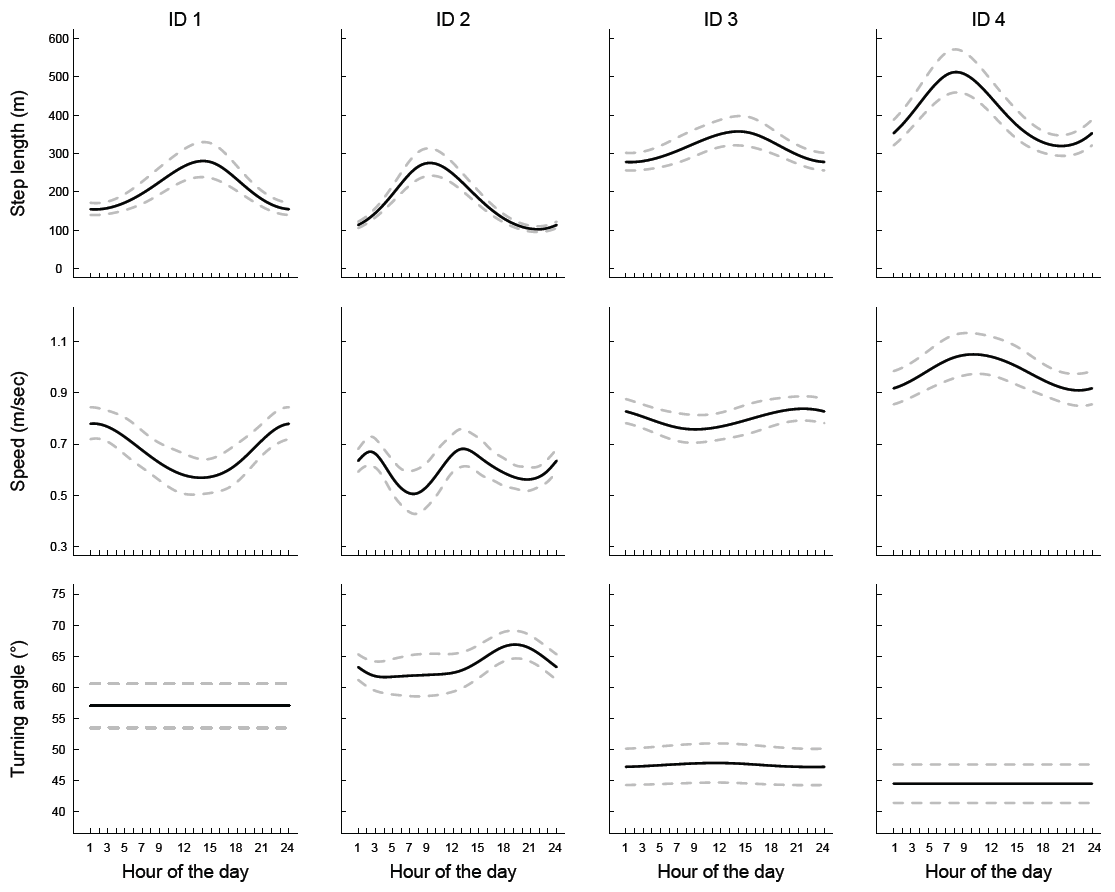


## Figure S4. Results of generalized additive mixed model (GAMM) analyses showing predicted (mean and 95% confidence intervals) effects of hour of the day on three horizontal movement parameters of four harbour porpoises captured in the inner Danish waters and fitted with a GPS-unit. To remove variation in natural behaviour from the data as much as possible but retain the unexplained and unpredictable variation in behaviour over time, we extracted the raw residuals (data minus fitted values) of each GAMM model and dependent variable (i.e. at the conditional or individual level) and calculated the mean value from all residuals within one-hour intervals over the complete tracking period. The model residuals were subsequently used to estimate the behavioural responses to capture/handling and airgun noise (see methods and results in main article). See Figure S6 for an overview of the full data used in the analyses.


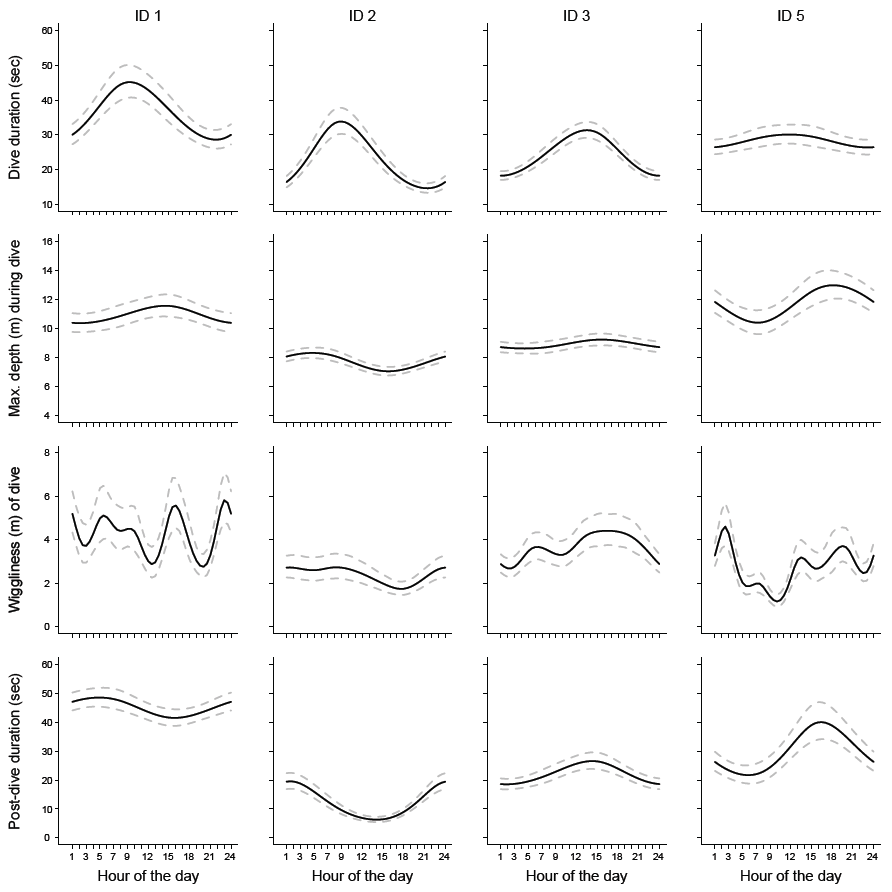


**Figure S5**. Results of generalized additive mixed model (GAMM) analyses showing predicted (mean and 95% confidence intervals) effects of hour of the day on four vertical movement parameters of four harbour porpoises captured in the inner Danish waters, and fitted with TDR-units. To remove variation in natural behaviour from the data as much as possible but retain the unexplained and unpredictable variation in behaviour over time, we extracted the raw residuals (data minus fitted values) of each GAMM model and dependent variable (i.e. at the conditional or individual level) and calculated the mean value from all residuals within one-hour intervals over the complete tracking period. The model residuals were subsequently used to estimate the behavioural responses to capture/handling and airgun noise (see methods and results in main article). See Figure S7 for an overview of the full data used in the analyses.


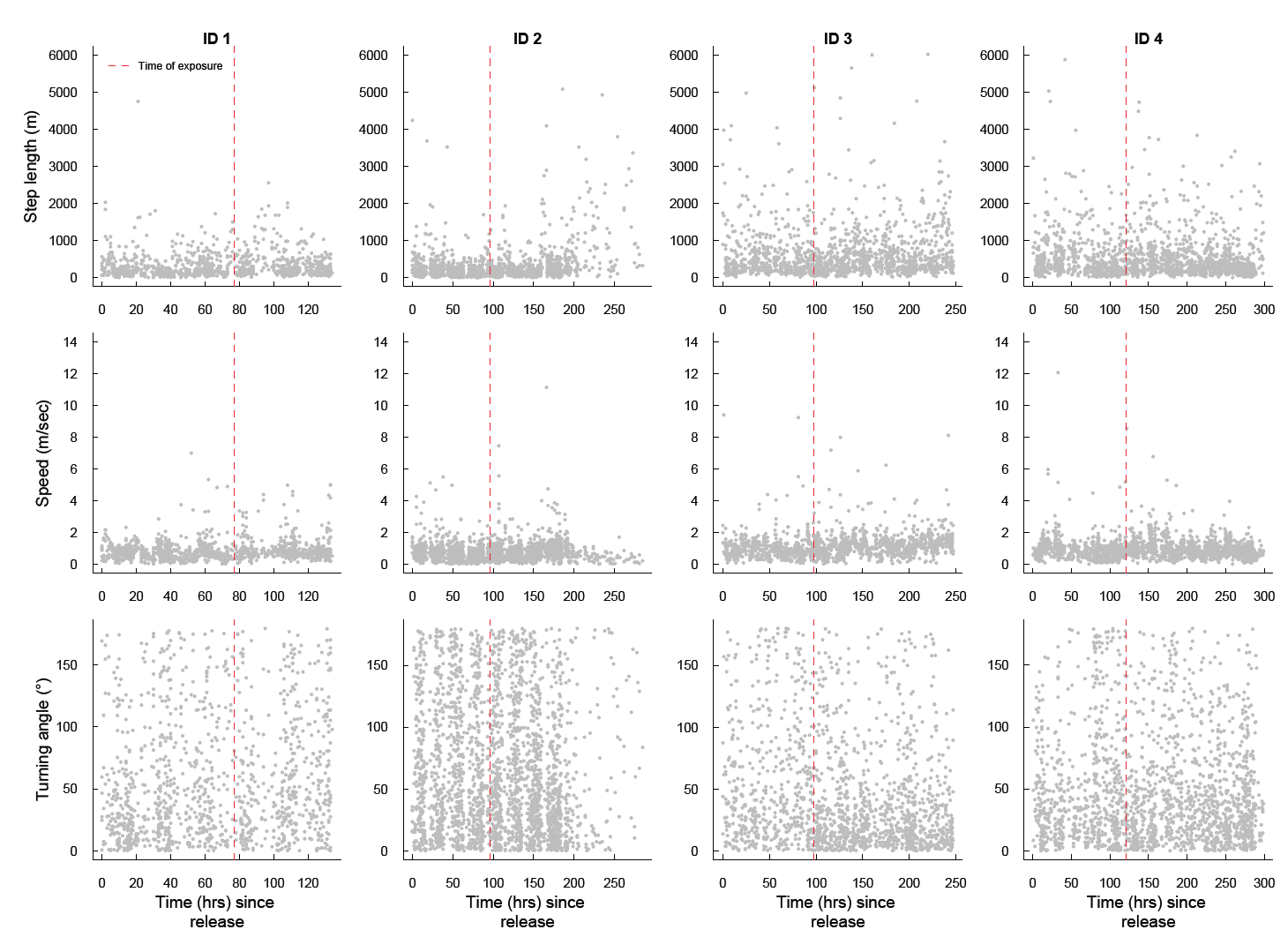


**Figure S6**. Overview of the full dataset for each horizontal movement parameters underlying the GAMM analyses in Figure S4.


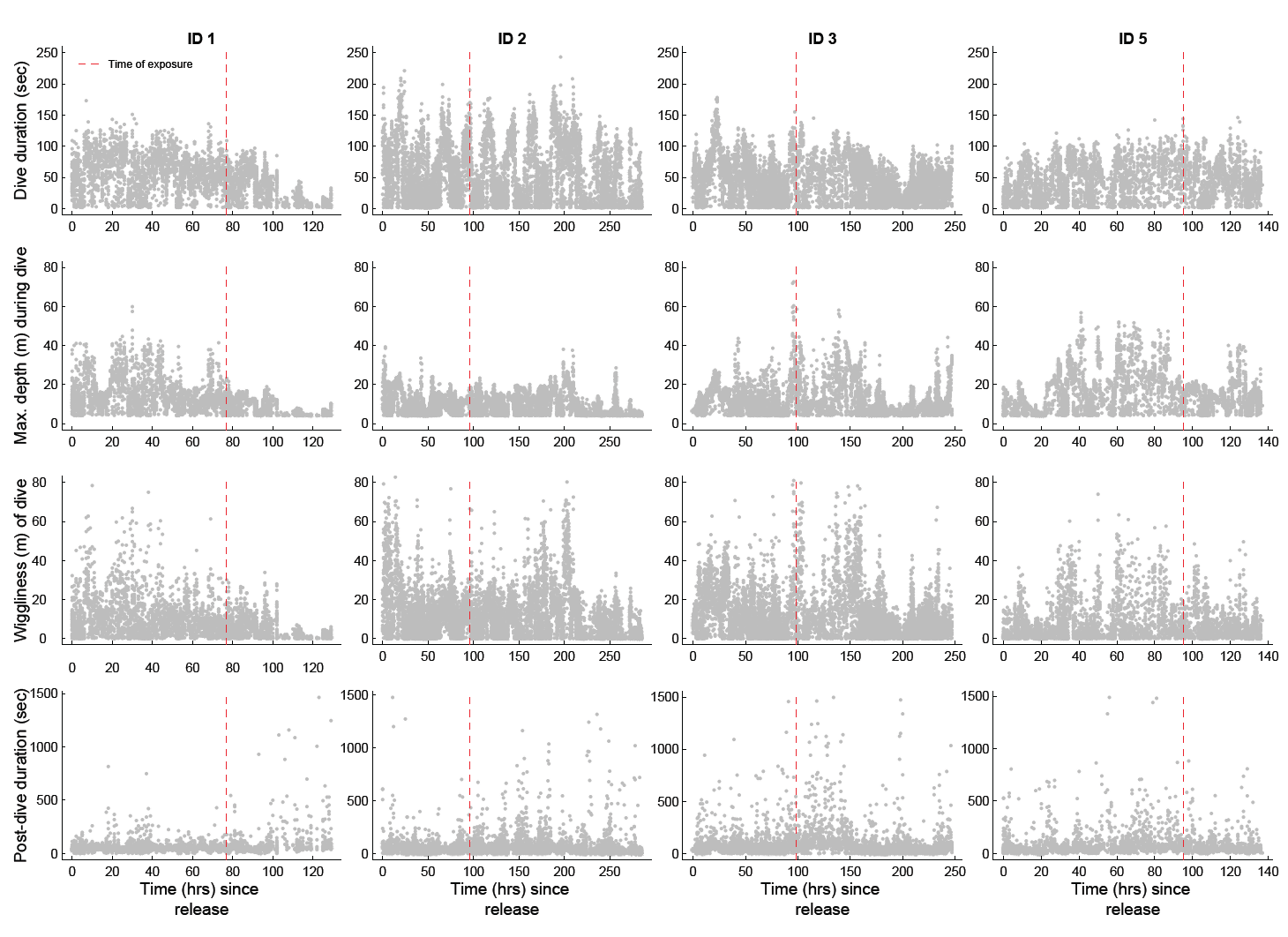


**Figure S7**. Overview of the full dataset for each vertical movement parameter underlying the GAMM analyses in Figure S5.


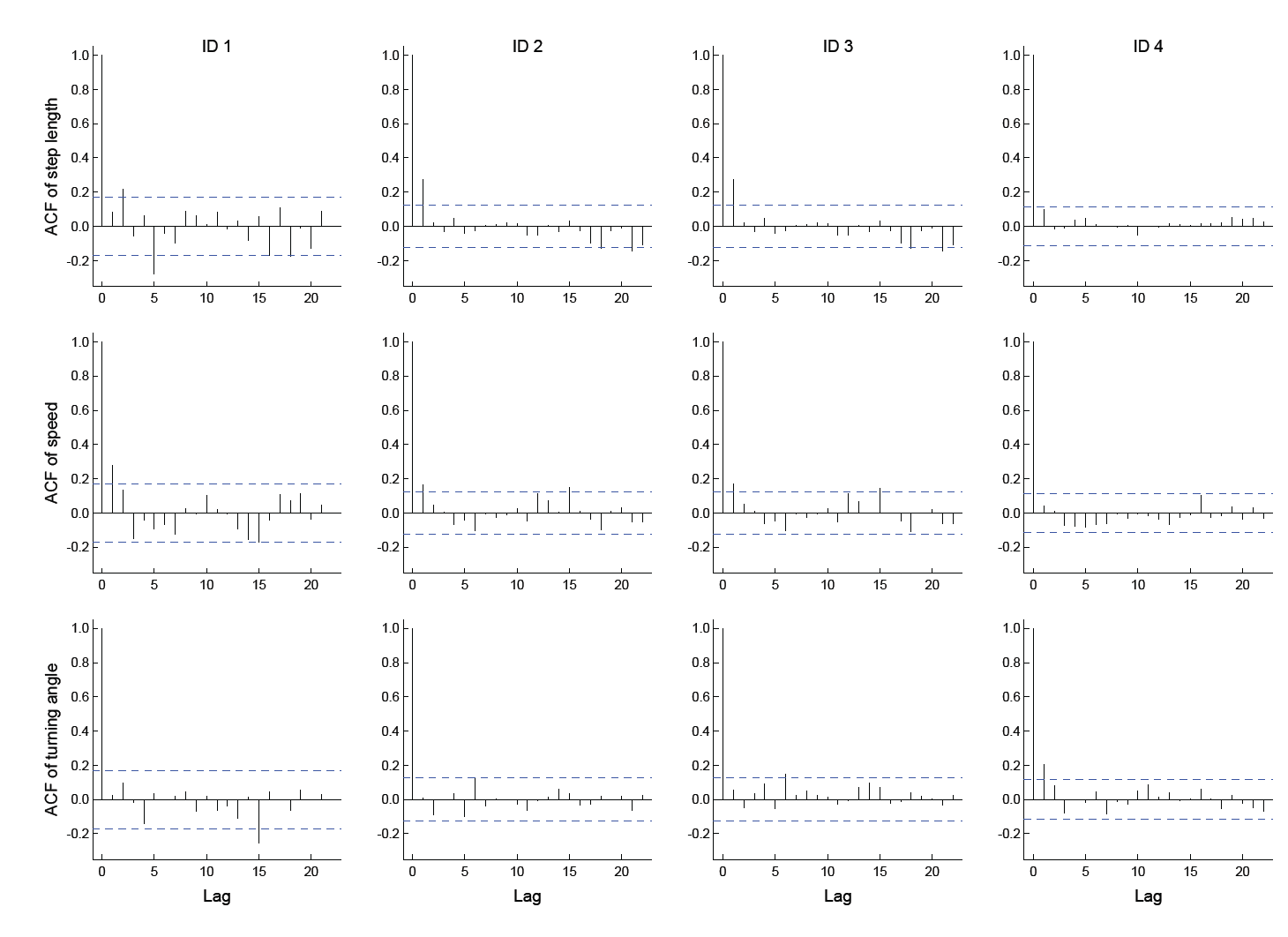


**Figure S8**. Overview of the temporal autocorrelation in piecewise regression model residuals of each horizontal movement parameter.


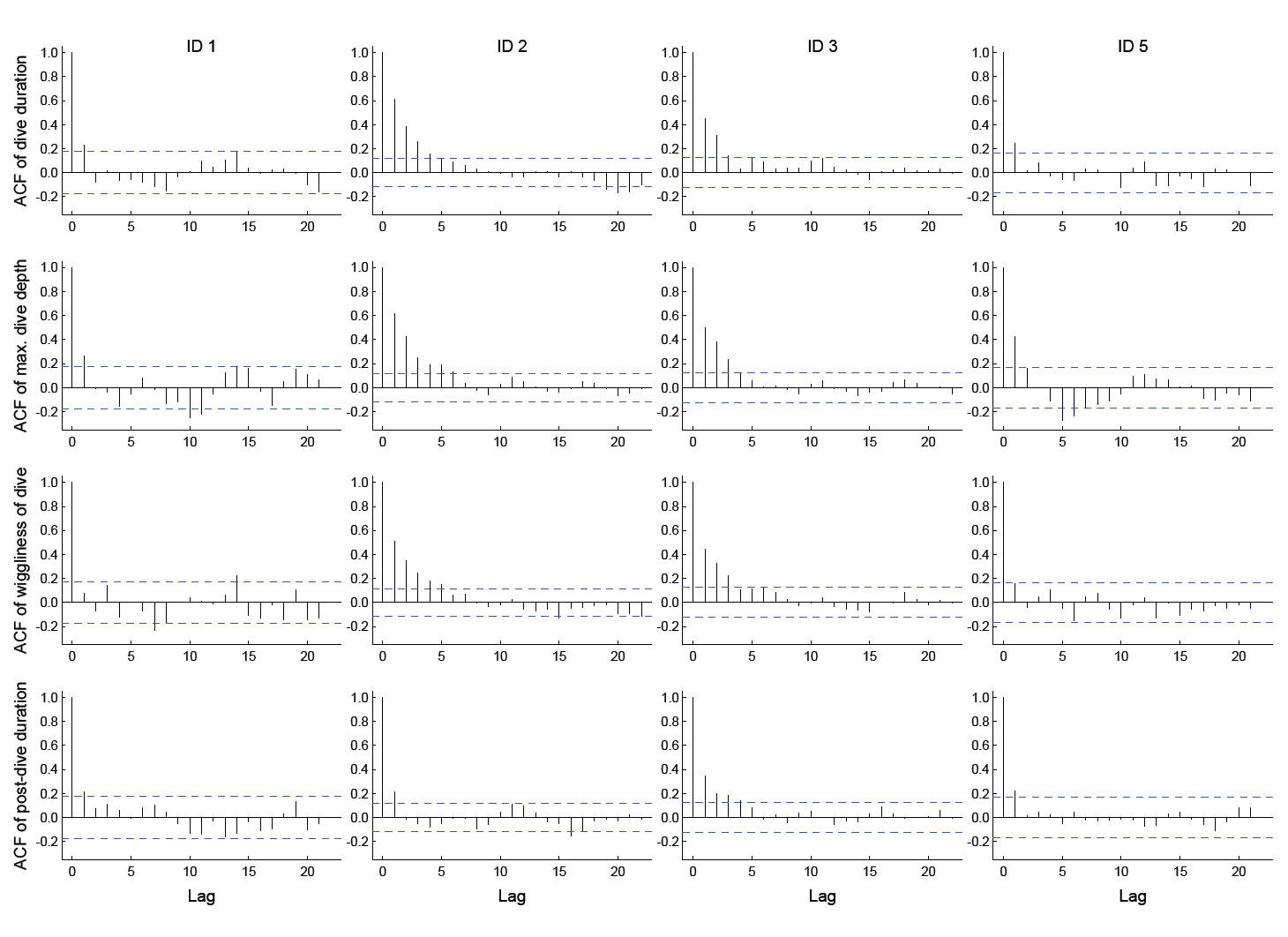


**Figure S9**. Overview of the temporal autocorrelation in piecewise regression model residuals of each vertical movement parameter.

**Table S1.** The mean (SE) values of breakpoints (h since release) as estimated by the best piecewise-regression models (based on AIC_c_) used to quantify timing of behavioural alterations in horizontal and vertical movement parameters of five harbour porpoises captured and tagged in the inner Danish waters and exposed to short-term noise pulses from a single airgun. Values for breakpoint 2 that are given in bold indicate that the breakpoint value aligned with the time of exposure (Table 1 in the main article). The coefficient (SE) of each piecewise regression line is provided in Table S2. The models form the analytical basis for Figs. 4−5 in the main article. Location data were missing for porpoise ID 5 and dive data were missing for porpoise ID 4 and as such changes in horizontal and vertical movement parameters respectively could not be analysed.

|  |  |  |  |  |
| --- | --- | --- | --- | --- |
| **Parameter/ID** | **Mean (SE) of breakpoints (h since release)** | | | |
| **Step length** | **1** | **2** | **3** | **4** |
| ID 1 | 8.01 (1.12) | - | - | - |
| ID 2 | 11.97 (3.28) | 144. 02 (9.46) | 273.31 (2.6) | - |
| ID 3 | 20.1 (2.53) | **98.34 (1.54)** | 102.1 (0.97) | 115.87 (2.38) |
| ID 4 | 10.26 (2.84) | - | - | - |
| **Speed** |  |  |  |  |
| ID 1 | 7.99 (1.64) | - | - | - |
| ID 2 | 20.21 (6.94) | 200.54 (15.54) | - | - |
| ID 3 | 20.11 (5.86) | **98.31 (2.32)** | 102.34 (1.59) | 135.05 (9.01) |
| ID 4 | 20.0 (5.11) | - | - | - |
| **Turning angle** |  |  |  |  |
| ID 1 | 35.9 (11.43) | - | - | - |
| ID 2 | 20.44 (15.74) | 150.1 (23.04) | 200.12 (9.86) | - |
| ID 3 | 10.43 (6.63) | **98.01 (2.05)** | 102.24 (1.43) | 135.66 (10.16) |
| ID 4 | 35.11 (9.85) | - | - | - |
| **Dive duration** |  |  |  |  |
| ID 1 | 7.63 (1.38) | 97.44 (2.4) | 122.1 (2.11) | - |
| ID 2 | 24.61 (4.17) | 183.9 (3.18) | 195.89 (4.81) | 221.03 (4.81) |
| ID 3 | 22.33 (2.46) | **98.02 (2.02)** | 105.29 (1.58) | 132.07 (5.04) |
| ID 5 | 10.63 (2.16) | **95. 61 (2.67)** | 103.01 (1.81) | 113.0 (2.83) |
| **Max dive depth** |  |  |  |  |
| ID 1 | 18.8 (2.28) | 89.42 (6.8) | 119.65 (4.99) | - |
| ID 2 | 20.05 (4.07) | 150.23 (9.17) | 190.46 (4.44) | 216.33 (8.512) |
| ID 3 | 22.77 (4.77) | **97.9 (3.67)** | 108.78 (2.81) | 122.7 (5.78) |
| ID 5 | 10.0 (2.95) | **95.25 (1.89)** | 103.22 (1.35) | 113.01 (2.62) |
| **Dive wiggliness** |  |  |  |  |
| ID 1 | 3.02 (9.09) | 92.62 (4.01) | 123.47 (3.04) | - |
| ID 2 | 27.54 (3.81) | 162.68 (9.27) | 197.44 (4.15) | 209.87 (6.62) |
| ID 3 | 21.31 (3.19) | 75.67 (7.33) | 157.24 (3.15) | 177.87 (3.34) |
| ID 5 | 20.00 (4.55) | - | - | - |
| **Post-dive duration** |  |  |  |  |
| ID 1 | 20.59 (5.05) | 90.98 (16.39) | 120.85 (16.28) | - |
| ID 2 | 12.07 (2.1) | - | - | - |
| ID 3 | 20.41 (5.04) | **98.15 (1.49)** | 105.28 (1.02) | 110.29 (1.22) |
| ID 5 | 20.32 (6.23) | **95.25 (1.82)** | 99.6 (1.36) | 113.35 (7.63) |

**Table S2.** The coefficient (SE) of each regression line as estimated by the piecewise-regression models to quantify timing of behavioural alterations in horizontal and vertical movement parameters of five harbour porpoises captured and tagged in the inner Danish waters and exposed to short-term noise pulses from a single airgun. The models form the analytical basis for Figs. 4−5 in the main article. Location data were missing for porpoise ID 5 and dive data were missing for porpoise ID 4 and as such changes in horizontal and vertical movement parameters respectively could not be analysed.

|  |  |  |  |  |  |
| --- | --- | --- | --- | --- | --- |
| **Parameter/ID** | **Coefficient (SE) of regression lines** | | | | |
| **Step length** | **1** | **2** | **3** | **4** | **5** |
| ID 1 | -1.37 (0.31) | 0.01 (0.006) | - | - | - |
| ID 2 | -0.77 (0.35) | -0.009 (0.001) | 0.09 (0.01) | -1.15 (0.47) | - |
| ID 3 | -0.46 (0.09) | 0.01 (0.01) | 1.99 (1.10) | -0.57 (0.18) | 0.001 (0.01) |
| ID 4 | -0.92 (0.49) | -0.009 (0.002) | - | - | - |
| **Speed** |  |  |  |  |  |
| ID 1 | -0.75 (0.24) | 0.009 (0.005) | - | - | - |
| ID 2 | -0.29 (0.16) | 0.006 (0.005) | -0.06 (0.02) | - | - |
| ID 3 | -0.122 (0.05) | -0.006 (0.007) | 0.74 (0.22) | -0.05 (0.02) | 0.005 (0.004) |
| ID 4 | -0.28 (0.13) | -0.001 (0.000) | - | - | - |
| **Turning angle** |  |  |  |  |  |
| ID 1 | 0.95 (0.31) | 0.05 (0.09) | - | - | - |
| ID 2 | 1.21 (0.54) | -0.01 (0.08) | -0.52 (0.24) | 0.83 (0.19) | - |
| ID 3 | 2.83 (1.30) | 0.24 (0.12) | -12.68 (5.92) | 1.11 (0.51) | 0.02 (0.08) |
| ID 4 | 0.94 (0.36) | -0.02 (0.02) | - | - | - |
| **Dive duration** |  |  |  |  |  |
| ID 1 | 0.82 (0.23) | -0.01 (0.06) | -0.31 (0.04) | 0.97 (0.32) | - |
| ID 2 | -0.25 (0.06) | 0.008 (0.001) | 0.43 (0.2) | -0.26 (0.06) | -0.02 (0.01) |
| ID 3 | 0.34 (0.05) | 0.01 (0.009) | -0.64 (0.22) | 0.15 (0.04) | 0.01 (0.004) |
| ID 5 | 0.53 (0.17) | 0.02 (0.07) | -0.52 (0.21) | 0.42 (0.19) | -0.04 (0.05) |
| **Max dive depth** |  |  |  |  |  |
| ID 1 | 0.17 (0.03) | -0.02 (0.05) | -0.08 (0.01) | 0.11 (0.13) | - |
| ID 2 | -0.11 (0.03) | -0.02 (0.002) | 0.038 (0.13) | -0.07 (0.02) | -0.02 (0.06) |
| ID 3 | 0.09 (0.03) | -0.003 (0.005) | -0.18 (0.09) | 0.09 (0.04) | 0.001 (0.002) |
| ID 5 | 0.21 (0.09) | 0.01 (0.004) | -0.39 (0.15) | 0.29 (0.11) | 0.02 (0.02) |
| **Dive wiggliness** |  |  |  |  |  |
| ID 1 | 0.32 (0.63) | -0.02 (0.01) | -0.41 (0.07) | 1.11 (1.15) | - |
| ID 2 | -0.41 (0.08) | 0.005 (0.008) | 0.16 (0.06) | -0.46 (0.22) | -0.08 (0.02) |
| ID 3 | 0.34 (0.08) | -0.06 (0.02) | 0.06 (0.01) | -0.35 (0.1) | 0.04 (0.01) |
| ID 5 | 0.30 (0.11) | -0.01 (0.008) | - | - | - |
| **Post-dive duration** |  |  |  |  |  |
| ID 1 | 0.84 (0.32) | -0.005 (0.05) | 0.23 (0.19) | -0.33 (1.41) | - |
| ID 2 | -2.23 (0.61) | 0.01 (0.06) | - | - | - |
| ID 3 | 0.56 (0.18) | 0.06 (0.02) | -2.99 (0.98) | 3.37 (1.65) | -0.007 (0.01) |
| ID 5 | 0.58 (0.23) | 0.06 (0.03) | -4.11 (1.97) | 0.82 (0.34) | 0.24 (0.19) |
